# Anatomy-Based Botulinum Toxin Injection of the Posterior Belly of Digastric Muscle

**DOI:** 10.3390/toxins18070302

**Published:** 2026-07-12

**Authors:** Hyun Jin Park, You-Jin Choi

**Affiliations:** Department of Anatomy, College of Medicine, Konkuk University, Chungju 27478, Republic of Korea; hjpark321@kku.ac.kr

**Keywords:** posterior belly of digastric muscle, Sihler’s staining, botulinum toxin, facial synkinesis, injection site

## Abstract

The posterior belly of the digastric muscle (PBDM) has recently been suggested as a target for botulinum toxin (BoNT) treatment in facial synkinesis. However, anatomically based injection sites for the PBDM have not been established. This study aimed to identify an ideal BoNT injection site by analyzing the intramuscular nerve distribution of the PBDM and its anatomical relationships with surrounding structures. Twelve PBDM specimens were obtained bilaterally from six embalmed cadavers. Modified Sihler’s staining was performed to visualize intramuscular nerve distribution. The PBDM was divided into anterior, middle, and posterior thirds, and the course and terminal branches of the facial nerve were analyzed. In all specimens, the PBDM was innervated by a single branch of the facial nerve. Nerve entry points were located in the middle third in 75% of cases and in the posterior third in 25%. Intramuscular nerve endings were most densely distributed in both the anterior and middle thirds in all specimens, whereas the posterior third showed a lower distribution (25%). The anterior and middle thirds of the PBDM appear to be the most appropriate targets for BoNT injection. The area 1 cm posterior to the angle of the mandible may serve as a practical landmark-guided injection site in clinical practice.

## 1. Introduction

Facial synkinesis is a representative facial movement disorder characterized by abnormal co-contraction, resulting not only in facial asymmetry but also in a wide range of functional discomfort. Although botulinum toxin (BoNT) injection is widely used in clinical practice, treatment has primarily focused on the superficial mimetic muscles and the platysma. However, in some patients, persistent tightness around the mandibular angle, deep aching pain, mastication-related discomfort, and swallowing-associated symptoms remain even after conventional treatment, suggesting that these symptoms cannot be fully explained by superficial facial muscles alone. Previous clinical studies have suggested that the posterior belly of the digastric muscle (PBDM) may represent an additional pathologic muscle in patients with refractory jaw discomfort, and symptom improvement has been reported following targeted chemodenervation of this muscle. These findings indicate that the PBDM may be considered not only a therapeutic target but also a diagnostically relevant muscle in the evaluation of deep perimandibular symptoms in patients with facial synkinesis [1].

The PBDM is located adjacent to the mandibular angle and contributes to mandibular depression, hyoid stabilization, and swallowing; accordingly, abnormal activation of this muscle may produce symptoms associated with jaw motion, mastication, and swallowing [1,2]. In previous clinical studies, patients with suspected PBDM involvement exhibited localized tenderness in the posteroinferior region of the mandibular angle, which was aggravated by mouth opening and neck flexion, and targeted injection was performed after localization of the deep PBDM using ultrasonography [1,2,3]. However, these approaches were based largely on clinical experience and imaging guidance, and no diagnostic or injection guideline has yet been established based on detailed intramuscular nerve distribution or precise anatomical relationships of the PBDM with surrounding structures [1,2,3,4,5,6].

The digastric muscle is composed of two bellies, anterior and posterior, which are joined by an intermediate tendon. The anterior belly originates from the digastric fossa of the mandible, whereas the posterior belly arises from the mastoid notch of the temporal bone, and they are innervated by the mandibular nerve and facial nerve, respectively [7,8]. Because the PBDM is located between the mastoid notch and the styloid process, it courses almost parallel to the stylohyoid muscle. Therefore, ultrasound-guided injections are strongly recommended to reliably distinguish these two muscles during the procedure [9,10,11]. While several studies have investigated the clinical significance of the anterior belly and suggested injection sites for injectable treatments [7,8,12,13], no studies have reported anatomically based injection sites for the PBDM. In previous studies, treatment of the PBDM was performed using ultrasound-guided or electromyography-guided injections [1,2,3]; however, these approaches cannot be regarded as evidence-based injection sites established on the detailed anatomy of PBDM.

BoNT acts by blocking the release of acetylcholine at nerve endings, thereby reducing muscle contraction. In addition to this well-established neuromuscular effect, BoNT-A has also been suggested to modulate the release of pain-related neurotransmitters, although the precise mechanism underlying its analgesic effect has not yet been fully clarified [14,15,16,17]. Because the action of BoNT depends on the nerve endings, accurate injection into the region where nerve endings are most densely distributed within the target muscle is expected to maximize the pharmacological effect while minimizing the required dose [18,19,20,21]. Therefore, precise localization of the intramuscular area with the highest density of nerve endings is a key anatomical consideration in establishing an effective injection strategy for BoNT procedures.

Manual dissection has inherent limitations in the evaluation of intramuscular nerve distribution. The dissection process may damage the muscle and alter the native course of fine intramuscular nerves. As a result, the original spatial arrangement of the intramuscular neural branches cannot be accurately maintained [22,23]. Sihler’s staining is a whole-mount nerve staining technique that renders muscle tissue translucent while selectively staining nerve axons. This technique is a specialized method for visualizing the intramuscular nerve patterns without manual dissection or deformity of the nerves. Although Sihler’s staining does not directly stain nerve endings, nerve endings are located at the distal ends of nerve axons. Evaluation of intramuscular nerve distributions can provide indirect evidence of regions where nerve endings are concentrated [24,25,26,27,28]. Therefore, the region with the highest density of nerve endings may represent the most appropriate injection site for BoNT procedures.

Sihler’s staining findings of the PBDM have not yet been reported. In this study, the intramuscular nerve distribution of the PBDM observed using Sihler’s staining. In addition, the anatomical relationships between the PBDM and the surrounding structures analyzed through manual dissection, with the aim of identifying the most ideal injection site for BoNT into the PBDM.

## 2. Results

### 2.1. Nerve Entry Points of PBDM

The number and location of nerve entry points to the PBDM were analyzed. In all cases (12/12), only one branch of the facial nerve entered the PBDM, and no specimen showed two or more entering branches. The entry points were identified in the middle third in 75% of cases (9/12) and in the posterior third in 25% of cases (3/12).

### 2.2. Densities of Nerve Endings in the PBDM

**Figure 1:**
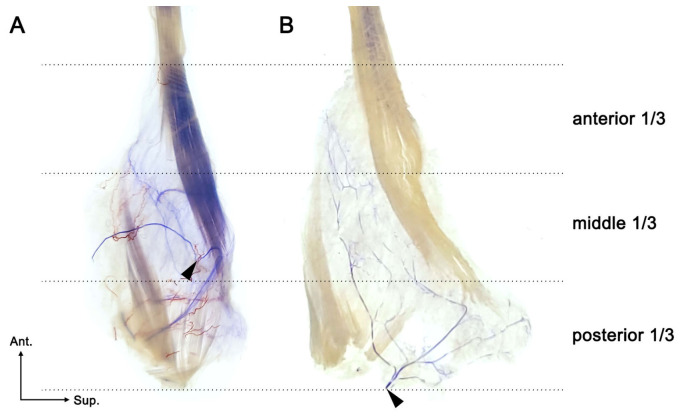
Results of Sihler’s staining of the posterior belly of the digastric muscle. All specimens are presented on the same scale. The stained neural structures, highlighted in purple, are primarily observed in both anterior and middle thirds. (**A**) Entry point in the middle third; (**B**) entry point in the posterior third; Arrowheads, indicate the entry points of the facial nerve.

The course and terminal branches of the facial nerve in each specimen were traced. The intramuscular nerve endings were most densely distributed in both the anterior and middle thirds of the PBDM (100%, 12/12 cases), followed by the posterior third (25%, 3/12 cases) (Figure 1).

## 3. Discussion

The digastric muscle is a major target for botulinum toxin injection. Because the anterior belly contributes to mandibular movement, various clinical applications of botulinum toxin injection into this muscle have been reported, including prevention of postoperative relapse after orthognathic or trauma surgery [29,30], treatment of sleep bruxism [8], and management of myogenic compression of the internal jugular vein [12]. In contrast, clinical reports on botulinum toxin injection into the PBDM remain very limited. The first study describing chemodenervation of the PBDM was reported by Pescarini et al. in 2021 [1], followed by a single subsequent study in 2023 [2]. Although further clinical reports on PBDM injections are expected in the future, the present study was designed primarily as an anatomical investigation aimed at identifying an accurate injection site rather than evaluating its clinical application.

Persistent tightness around the mandibular angle, deep aching pain, and discomfort during mastication or swallowing cannot always be explained by abnormalities of the superficial mimetic muscles or platysma alone. Previous clinical studies have reported symptomatic improvement after targeted chemodenervation of the PBDM in patients with these refractory symptoms. In addition, high-resolution ultrasonography has been used to localize deep muscles in facial synkinesis [1,2], but detailed anatomical criteria for the PBDM have not yet been established. Therefore, the present findings may assist in identifying PBDM involvement, distinguish it from more superficial muscle involvement, and support more precise ultrasound-based localization and selective injection planning.

This study demonstrated the intramuscular nerve distributions of the PBDM. Sihler’s staining is a useful method for visualizing nerve endings without manual dissection or nerve deformities. In the present study, most nerves entered the PBDM in the middle third (75%, 9/12 cases), and nerve endings were observed in both the anterior and middle thirds in all specimens (100%, 12/12 cases) and the posterior third in a smaller proportion of cases (25%, 3/12 cases). These findings suggest that the areas with dense nerve endings may serve as useful anatomical guidelines for identifying effective BoNT injection sites in PBDM.

**Figure 2:**
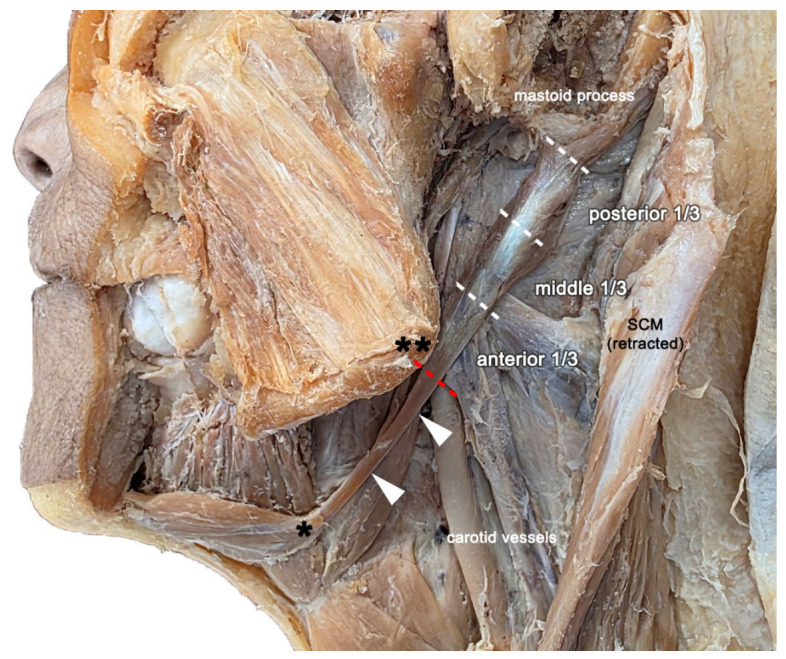
Dissected posterior belly of the digastric muscle (PBDM). The parotid gland was removed, and the sternocleidomastoid muscle was retracted to expose the PBDM. Arrowheads, intermediate tendon; red dotted line, boundary between tendinous and muscular portion of PBDM; *, hyoid bone; **, angle of mandible.

An accurate understanding of the anatomical structure of the PBDM is essential when determining an appropriate injection site. The digastric muscle is composed of two muscular bellies joined by an intermediate tendon. The PBDM originates from the mastoid notch of the temporal bone and extends to the intermediate tendon, which is anchored to the hyoid bone by a fibrous sling. Importantly, because only the tendon is present at the proximal end of the PBDM, this area should be excluded from the injection target. Our anatomical dissection showed that the boundary between the tendon-only portion and the muscular portion approximately corresponds to the level of the angle of the mandible. Therefore, the area between the hyoid bone and the angle of the mandible contains only the tendinous portion of the PBDM. Because both structures are easily palpable surface landmarks, clinicians will be easily able to determine the tendinous portion of PBDM in clinical field (Figure 2).

A previous study on chemodenervation of the PBDM designated the injection point as 1 cm inferior and posterior to the angle of the mandible [1]. However, the area located 1 cm inferior to the angle of the mandible is in close proximity to the carotid vessels. Even with ultrasound-guided injections, if the needle is advanced slightly deeper than intended, there is a substantial risk of vascular injury. In addition, this area corresponds to the boundary between the tendinous and muscular portions of the PBDM, where the muscular component is relatively limited. Therefore, it is necessary to establish a new injection site within the muscular portion of the PBDM that shows rich nerve distributions and is located away from critical structures such as the carotid vessels (Figure 2).

Sihler’s staining demonstrated that intramuscular nerve endings of the PBDM were consistently distributed in both the anterior and middle thirds in all specimens. These findings suggest that BoNT injections should target the anterior and middle thirds of the PBDM. In contrast, the posterior third is considered a less suitable injection site because (1) ultrasound transducer placement is limited in this region by the earlobe; (2) this portion lies deep beneath the mastoid process and sternocleidomastoid muscle; and (3) nerve endings were relatively sparse in this region (25%).

**Figure 3:**
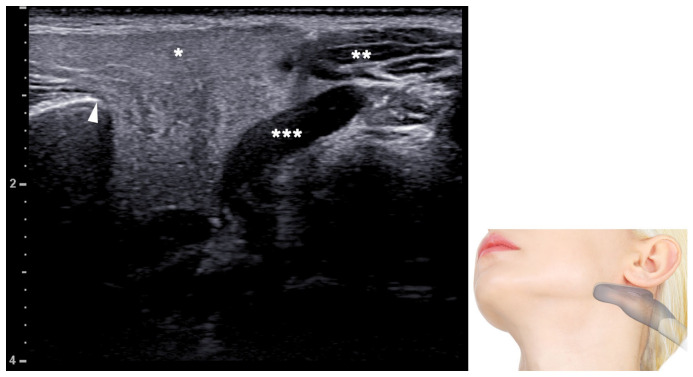
Ultrasonographic image of the region posterior to the angle of the mandible. An ultrasound scanner (HS70A; Samsung, Seoul, Republic of Korea) with a linear transducer (L3-12A) was used in this study. Arrowhead, angle of mandible; *, parotid gland; **, sternocleidomastoid muscle; ***, posterior belly of digastric muscle. Adapted from data provided by H.J. Kim, with permission [8].

The area located 1 cm posterior to the angle of the mandible corresponds to the anterior or middle third of the PBDM. On ultrasonography, the parotid gland is identified in this region, with the PBDM located at a deeper plane. Therefore, access to the PBDM in this area requires passage through the parotid gland, which may represent potential side effects. However, the region posterior to the angle of the mandible corresponds to the tail of the parotid gland [31,32,33], rather than the course of the main trunk of the facial nerve, and major vessels such as the facial artery and carotid artery do not traverse this area. Therefore, this region may be considered a relatively safe injection site for PBDM (Figure 3).

Although variations in the digastric muscle have been reported more frequently in the anterior belly, several anatomical variations have also been described in the PBDM. Reported variations in the PBDM include duplication or a double posterior belly, a bifid origin, and complete absence [5,34,35]. These variations are clinically relevant because the PBDM serves as an important anatomical landmark in the upper neck and parotid region, and failure to recognize such variants may complicate surgical dissection or alter the expected location of the injection target. In the 12 specimens examined in the present study, no such morphological variations were identified. Nevertheless, the possibility of PBDM variation should be considered when planning botulinum toxin injection, and ultrasonographic confirmation may further improve procedural accuracy and safety.

Ultrasonography is useful for localizing the PBDM and identifying adjacent critical structures during the procedure. The injection site proposed in the present study is located between the angle of the mandible and the anterior border of the sternocleidomastoid muscle, approximately 1 cm posterior to the angle of the mandible. At this location, Doppler mode can be used to readily identify the presence of major vessels, such as the external jugular vein and carotid artery. Therefore, ultrasonography should be used not only to ensure accurate needle placement into the PBDM, but also to evaluate the real-time relationships among adjacent muscles, the parotid gland, and vascular structures so that the procedure can be performed more safely.

Therefore, ultrasound-guided injections are strongly recommended to ensure selective targeting of the PBDM. If the injection is placed too superficially and enters the parotid gland, salivary dysfunction may occur. If the injection is placed too deeply and enters the stylohyoid muscle, dysphagia may develop because of impaired elevation of the hyoid bone and larynx. In addition, if botulinum toxin is delivered extramuscularly, it may affect not only adjacent muscles but also more distant muscles [36]. Furthermore, the present study did not provide depth information from the skin surface to the PBDM, because even when the needle is entered at the proposed injection site, blind injection technique cannot ensure accurate access to the PBDM. Therefore, ultrasound guidance injections are essential to ensure accurate intramuscular injections.

Several limitations should be acknowledged. First, the injection dose was not addressed in the present study. Dose adjustment according to muscle size is an important factor for successful treatment. However, this study primarily focused on identifying an accurate injection site specifically targeting the PBDM. Second, the sample size was relatively small. 12 specimens may not be sufficient to generalize the intramuscular nerve distribution patterns of the PBDM. In addition, because only Korean cadavers were used, the findings of this study do not reflect potential racial differences.

## 4. Conclusions

This study demonstrated the intramuscular nerve distributions of the PBDM using Sihler’s staining and identified the anterior and middle thirds as the regions with the highest density of intramuscular nerve endings. In contrast, the proximal tendinous portion should be excluded from the injection target, and the posterior third appears to be a less favorable target because of its limited nerve distributions and difficult anatomical accessibility. Based on these findings, the area 1cm posterior to the angle of the mandible may serve as an easy guideline for targeting the anterior or middle third of the PBDM. Because this study was focused on anatomical findings, further studies are needed to validate clinical applicability. In particular, the appropriate injection dose according to muscle size should be investigated. As the optimal dose may vary according to sex, individual characteristics, and ethnicity, such studies may contribute to the establishment of a more precise injection guideline.

The principal strength of the present study lies in providing the first Sihler’s staining-based intramuscular nerve distribution patterns of the PBDM, thereby suggesting an anatomical-based injection point. In addition, this study not only described the neural distribution of the PBDM, but also examined its anatomical relationships with adjacent structures through cadaveric dissection. Palpable surface landmarks, such as the angle of the mandible, were linked to a clinically applicable injection site. The applicability of ultrasonography was also discussed, thereby integrating anatomy, procedure, and imaging into a single clinical framework. In this respect, the present study provides a meaningful anatomical basis for the development of a more precise botulinum toxin injection. Nevertheless, further studies are needed before these anatomical findings can be applied in clinical practice.

## 5. Materials and Methods

Six embalmed Korean cadavers (3 males and 3 females; mean age, 78.5 years; range, 69–88 years) were used in this study. The PBDMs were obtained bilaterally from all cadavers. This cadaveric study was approved by the institutional review board of Konkuk University on 23 February 2026 (KKUIRB-202603-BR-190). None of the cadavers showed any gross pathology or underwent surgical procedures in the examined area. The skin, subcutaneous tissue, and platysma in the neck region were removed in a layer-by-layer manner. To fully expose the PBDM, the parotid gland was completely removed, and the sternocleidomastoid muscle was partially retracted to clearly delineate the digastric muscle. The common carotid artery and internal jugular vein adjacent to the PBDM were also carefully dissected to evaluate their anatomical relationships with the muscle. The digastric muscle was then harvested intact by detaching its attachments from the mandible and mastoid notch.

### Sihler’s Staining

Sihler’s staining consists of seven steps and generally requires 3–4 months to complete. In this technique, both the muscle and nerves are initially stained, after which the neural structures are preserved while only the muscle and subcutaneous tissue are selectively destained, allowing visualization of the intramuscular nerve distribution. In this study, a modified Sihler’s staining technique was used to demonstrate the intramuscular nerve distribution of the PBDM. The detailed staining procedure was as follows [10,22,23,24,25,26,27,28].

**Fixation** The harvested PBDM specimens were fixed in 10% neutral formalin (DUKSAN, Ansan-si, Republic of Korea) for at least 4 weeks. This step was performed to prevent tissue autolysis and to preserve the nerve sheath.

**Maceration and depigmentation** The specimens were placed in 3% potassium hydroxide (DAEJUNG, Siheung-si, Republic of Korea) solution for maceration and depigmentation. This process continued until the specimens became semitransparent and the nerves could be identified clearly as fibrous whitish structures.

**Decalcification** The specimens were placed in Sihler’s solution I (glacial acetic acid (DAEJUNG, Siheung-si, Republic of Korea) 1/8%, glycerin (DAEJUNG, Siheung-si, Republic of Korea) 1/8%, 1% aqueous chloralhydrate (DAEJUNG, Siheung-si, Republic of Korea) 6/8%). The solution was refreshed weekly.

**Staining** Following decalcification step, the specimens undergo in Sihler’s solution II (Ehrlich’s hematoxylin (DAEJUNG, Siheung-si, Republic of Korea) 1/8%, glycerin 1/8%, 1% aqueous chloralhydrate 6/8%). Staining continued until all nerve branches were stained dark blue to violet.

**Destaining** The destaining procedure was performed by returning the specimens to Sihler’s solution I for approximately 2–8 h. This step was carefully monitored until the nerve branches retained a dark blue or violet color, whereas the surrounding non-neural tissues became transparent.

**Neutralization** The specimen must be neutralized for 1 h in a 0.05% lithium carbonate (DAEJUNG, Siheung-si, Republic of Korea) solution. Washing under running water for 1 h is required before the clearing stage.

**Clearing and preservation** The specimens were first placed in 40% glycerin prepared by mixing glycerin and distilled water at a ratio of 4:6, followed sequentially by 60% glycerin (6:4) and 80% glycerin (8:2). Finally, the specimens were placed in 100% glycerin to complete the clearing and preservation process. Each concentration was maintained for 1 day.

**Figure 4:**
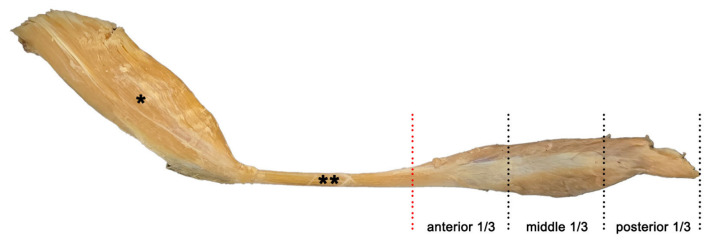
Three regions of the posterior belly of the digastric muscle. The specimens were divided into three equal parts defined as anterior 1/3, middle 1/3, and posterior 1/3 excluding the intermediate tendon. Red dotted line, boundary between tendinous and muscular part of the PBDM; *, anterior belly of digastric muscle; **, intermediate tendon.

The stained specimens were carefully trimmed on an illuminated viewing box to allow clear visualization of the intramuscular nerve pathways. The course of the nerves within the PBDM was documented by photography and schematic drawing. Then, the regional distribution of the intramuscular nerve endings was evaluated. To present our findings more effectively, the PBDM was divided into three equal regions, defined as the anterior 1/3, middle 1/3, and posterior 1/3 (Figure 4).

## Data Availability

The original contributions presented in this study are included in the article. Further inquiries can be directed to the corresponding author.

## Abbreviations

The following abbreviations are used in this manuscript:

PBDM	Posterior Belly of Digastric Muscle
BoNT	Botulinum Toxin
